# Discovery and Functional Characterization of Novel Aquaporins in Tomato (*Solanum lycopersicum*): Implications for Ion Transport and Salinity Tolerance

**DOI:** 10.3390/cells14171305

**Published:** 2025-08-22

**Authors:** Newton Chandra Paul, Shahin Imran, Anri Mitsumoto, Izumi C. Mori, Maki Katsuhara

**Affiliations:** 1 Institute of Plant Science and Resources, Okayama University, Kurashiki 710-0046, Japan; p7rq8hrw@s.okayama-u.ac.jp (N.C.P.); ptj87a5q@s.okayama-u.ac.jp (S.I.); imori@okayama-u.ac.jp (I.C.M.); 2 Department of Agronomy, Khulna Agricultural University, Khulna 9100, Bangladesh

**Keywords:** Aquaporin (AQP), ion transport, plasma membrane intrinsic proteins (PIPs), tomato, oocytes, water transport

## Abstract

Aquaporins (AQPs) are membrane proteins that facilitate the transport of water and solutes. Among AQPs, plasma membrane intrinsic proteins (PIPs) play a critical role in maintaining water balance between the internal and external cell environments. This study focuses on the tomato due to its economic importance and cultivation under moderate salinity conditions in Japan. A swelling assay using *X. laevis* oocyte confirmed that all five examined tomato SlPIP2 isoforms showed water transport activity. Among them, two-electrode voltage clamp (TEVC) experiments showed that only SlPIP2;1, SlPIP2;4, and SlPIP2;8 transport Na^+^ and K^+^, with no transport activity for Cs^+^, Rb^+^, Li^+^, or Cl^−^. CaCl_2_ (1.8 mM) reduced ionic currents by approximately 45% compared to 30 µM free-Ca^2+^. These isoforms function as very low-affinity Na^+^ and K^+^ transporters. Expression analysis showed that *SlPIP2;4* and *SlPIP2;8* had low, stable expression, while *SlPIP2;1* was strongly upregulated in roots NaCl treatment (200 mM, 17days), suggesting distinct physiological roles for these ion-conducting AQPs (icAQPs). These data hypothesized that tomato icAQPs play a critical role in ion homeostasis, particularly under salinity stress. In conclusion, the first icAQPs have been identified in the dicotyledonous crop. These icAQPs are essential for plant resilience under salt stress.

## 1. Introduction

Aquaporins (AQPs) are membrane proteins that primarily facilitate water movement but are also capable of transporting ions, gases, and small molecules [1,2]. In plants, AQPs belong to the major intrinsic protein (MIP) superfamily and play a vital role in maintaining physiological homeostasis [3]. These AQPs are grouped into five subfamilies [4]. Among them, plasma membrane intrinsic proteins (PIPs) have a critical role in water transport and maintaining osmotic balance under salt stress [3,5]. Recent research indicates that some AQPs can also function as non-selective cation channels (NSCCs) [6], which contribute to salt tolerance [7]. PIPs are further divided into PIP1 and PIP2 subgroups [8]; some serve exclusively as water channels, while others can also transport different solutes, highlighting their diverse functions in plant physiology. In *Arabidopsis*, AtPIP2;1 and AtPIP2;2 have been shown to mediate non-selective cation conductance, primarily involving Na^+^, when expressed in *Xenopus laevis* oocytes [6,9]. Similarly, the barley aquaporin HvPIP2;8 has been identified as an NSCC, mainly transporting Na^+^ and K^+^ in *X. laevis* oocytes [10]. HvPIP2;8 functions as a Ca^2+^-sensitive aquaporin in barley, upregulating in shoot tissues under salt stress, and contributing to salt stress adaptation [10]. Consistently, a two-electrode voltage clamp (TEVC) study in *X. laevis* oocytes identified OsPIP2;4 as a strong cation-conducting aquaporin that facilitates Na^+^ and K^+^ transport, reinforcing its classification as an ion-conducting AQP (icAQP) with a potential dual role in water and cation transport [11].

In recent years, there has been a growing interest in plant AQPs, particularly regarding their functions in response to abiotic stresses such as salinity, drought, and cold [12]. In rice, AQPs contribute substantially to root hydraulic conductivity, accounting for up to 79% under well-watered conditions, and increasing to 85% during drought stress [13]. In chrysanthemum (*Dendranthema morifolium*), overexpression of *CmPIP1* and *CmPIP2* significantly improved salt tolerance in transgenic lines [14]. Similarly, the *MaPIP1–1* gene from *Musa nana* enhanced salt tolerance when expressed in *Arabidopsis* [15]. Transgenic *Arabidopsis* plants overexpressing maize *ZmPIP1–1* showed increased resistance to both drought and salinity stress [16].

In contrast to the well-studied role of AQPs in monocot crops (such as rice and barley), the ion transport function of AQPs in dicot crops remains unexplored. Although icAQPs were previously reported in the dicot *Arabidopsis* [6,9], it is not a crop species, and its value for applied crop improvement research is limited due to its naturally low salt tolerance and lack of essential agronomic traits related to yield [17]. Therefore, we focused on the tomato (*Solanum lycopersicum* L.), a widely cultivated and economically important dicot vegetable crop [18]. Moreover, as a moderately salt-sensitive species, it provides a relevant system for studying salt tolerance in an agriculturally meaningful context [19]. Tomato is valued not only as fresh produce but also as a model plant for studying genetics, fruit development, and stress responses [19,20]. Salt stress has been shown to impact tomato plant growth and fruit yield adversely [21]. Exposure to salinity leads to excessive Na^+^ uptake by the roots, which lowers the osmotic potential and restricts water absorption, ultimately inhibiting root development [22,23]. In the short term, salt stress also reduces stomatal conductance, stomatal pore size, and stomatal index, significantly limiting photosynthetic activity in leaves [24]. Recent studies showed that some AQPs play a crucial role in coping with salt stress through various molecular mechanisms [12]. In this study, the tomato PIP2 genes SlPIP2;1, SlPIP2;4, SlPIP2;6, SlPIP2;8, and SlPIP2;9 were examined for their ion transport activity using TEVC experiments in *X. laevis* oocytes in the present study. The results showed that SlPIP2;1, SlPIP2;4, and SlPIP2;8 exhibited cation conductance in addition to the water transport activity, thus qualifying them as icAQPs. Their ion transport characteristics (inhibitory effects of divalent cations, alkali cation selectivity sequence, as well as the affinity for Na^+^ and K^+^), and expression patterns in roots, leaves, and stems are investigated. These findings provide new insights into the roles of these icAQPs in ion homeostasis and contribute to a deeper understanding of the mechanisms underlying salt tolerance in tomato plants.

## 2. Materials and Methods

### 2.1. Plant Materials and Growth Conditions

Seedlings of *Solanum lycopersicum* cv. Micro-Tom were cultivated in Metromix soil under controlled conditions (25 °C, 60% relative humidity, 120 µmol s^−1^ m^−2^, 16 h light/8 h dark; Biotron LPH200, Nippon Medical & Chemical Instruments, Osaka, Japan) as described previously [25]. Plants were watered twice weekly and fertilized once every two weeks with a 500-fold dilution of Hyponex concentrate (Hyponex, Osaka, Japan).

### 2.2. RNA Extraction and cDNA Synthesis

Total RNA was extracted from leaves of 6-week-old plants using the RNeasy Plant Mini Kit (QIAGEN, Hilden, Germany) and used to synthesize first-strand cDNA with the PrimeScript II 1st strand cDNA Synthesis Kit (TaKaRa, Shiga, Japan). Gene-specific primers (Supplementary Table S1) were designed using Primer3 based on published sequences to amplify the full-length coding sequences of *SlPIP2;1*, *SlPIP2;4*, *SlPIP2;6*, *SlPIP2;8*, and *SlPIP2;9* using Ex Taq polymerase (TaKaRa). PCR products were cloned into the pGEM-T Easy Vector (Promega, Madison, WI, USA) by TA cloning.

### 2.3. Preparation of SlPIP cRNAs

Full-length coding sequences were subcloned into the BglII site of the pXβG-ev1 vector, which contains *X. laevis* β-globin UTRs to enhance mRNA stability and translation, using a one-step PCR method [26,27]. Primer sequences used for subcloning are listed in Supplementary Table S2. The constructs were then linearized, and cRNAs were synthesized using the mMESSAGE mMACHINE T3 kit (Ambion, Austin, TX, USA) at a final concentration of 1 µg/µL.

### 2.4. Expression of SlPIP2s in X. laevis Oocytes

Oocytes were collected from adult female *X. laevis* frogs, thoroughly washed, and placed in modified Barth’s solution (MBS). The MBS contained 88 mM NaCl, 1 mM KCl, 2.4 mM NaHCO_3_, 1.5 mM Tris-HCl (pH 7.6), 0.3 mM Ca(NO_3_)_2_·4H_2_O, 0.41 mM CaCl_2_·4H_2_O, and 0.82 mM MgSO_4_·7H_2_O, supplemented with 10 µg/mL penicillin sodium salt and 10 µg/mL streptomycin sulfate. Each oocyte was microinjected with 10 ng of *SlPIP2* cRNA or nuclease-free water (control) and then incubated at 18 °C for 24–48 h before undergoing electrophysiological and swelling assays [28].

### 2.5. Water Swelling Assays

Oocytes injected with either *SlPIP2s* cRNA or water were incubated in MBS at 18 °C for approximately 24–48 h. Following incubation, they were transferred from the 1-fold MBS (200 mosmol Kg^−1^) to a 1/5-fold diluted MBS (40 mosmol Kg^−1^) to assess water influx through swelling assays. Water permeability coefficient (*P*_f_) was determined by measuring changes in the cell volume of each oocyte. Cell volume changes were monitored using an inverted microscope equipped with a monochrome digital camera (Cool SNAP, Roper Scientific, Tucson, AZ, USA). Images were taken every 10 s over 90 s using MetaVue software (version 7.5, Molecular Devices Analytical Technologies, Downingtown, PA, USA). The oocyte surface area was subsequently measured using WinRoof software (version 3.51, MITANI Corporation, Fukui, Japan), and the volume change was estimated.

### 2.6. Electrophysiology

TEVC experiments were performed using *X. laevis* oocytes microinjected with either *SlPIP2* cRNA or nuclease-free water. Borosilicate glass pipettes (Harvard Apparatus, GC150TF-10; 1.5 mm O.D. × 1.17 mm I.D.) were pulled and filled with 3 M KCl for use as voltage and current injection electrodes. To minimize the effects of series resistance in the bath solution, a bath clamp system was employed. Bath current and voltage sensing electrodes were composed of silver–silver chloride electrodes connected to the bath via 3% agar bridges containing 3 M KCl and were employed as previously described [10]. Bath solutions were prepared with either high external calcium (1.8 mM MgCl_2_, 1.8 mM mannitol, 1.8 mM CaCl_2_, 10 mM HEPES; pH 7.5 adjusted with Tris) or low external calcium (1.8 mM MgCl_2_, 1.8 mM EGTA, 1.8 mM CaCl_2_, 10 mM HEPES; pH 7.5 adjusted with Tris), unless otherwise specified. Osmolality was maintained at 200 mOsm/kg by adjusting with mannitol. Divalent cations (1.8 mM Ca^2+^ and 1.8 mM Mg^2+^) and monovalent cations (96 mM Na^+^, K^+^, Li^+^, Cs^+^, and Rb^+^) were introduced as chloride or gluconate salts. TEVC recordings were conducted 24–36 h post-injection. Recordings were performed and analyzed using an Axoclamp 900A amplifier (Molecular Devices, San Jose, CA, USA) and Clampex 9.0 software (Molecular Devices, San Jose, CA, USA) at room temperature (20–22 °C). Biological replicates consisted of multiple oocytes obtained from different frogs and batches, with representative results from two or more oocyte batches per experiment unless otherwise mentioned, as shown in the figures.

Conductance measurements were obtained across the tested concentrations at membrane potentials ranging from −75 to −120 mV. Ion permeability ratios were calculated from shifts in the reversal potential using a modified Goldman equation, as previously described [11], which ignored Cl^−^ permeability and considered only monovalent cations such as Na^+^, K^+^, Rb^+^, Cs^+^, and Li^+^.

### 2.7. Gene Expression Analysis Using qPCR

Tomato plants were grown in soil-filled pots under controlled conditions (25 °C, 60% relative humidity, 120 µmol s^−1^ m^−2^, 16 h light/8 h dark) for approximately 30 days before being divided into two treatment groups. Three plants were irrigated with 200 mM NaCl in tap water, while the other three received only tap water for 17 days. At around 47 days of age, leaf, root, and stem samples were collected from the plants and analyzed (Supplementary Figure S1). The samples were rinsed and immediately frozen in liquid nitrogen. Total RNA was extracted using a mortar and pestle along with the RNeasy Plant Mini Kit (Qiagen, Hilden, Germany). cDNA synthesis was performed using the High-Capacity cDNA Reverse Transcription Kit (Applied Biosystems by Thermo Fisher Scientific). For the absolute quantification of transcript copy numbers, the short specific sequence of the genes was cloned into the PCR4 TOPO vector by TA cloning (Invitrogen, Carlsbad, CA, USA) and then subcloned into the BglII site of the pXβG-ev1 vector [26,27]. After that, cRNAs were produced using the mMESSAGE mMACHINE T3 kit (Ambion, Austin, TX, USA). A series of known concentrations of these cRNAs (ranging from 10^8^ to 10^12^ copies, based on their size and molecular weight) was prepared by serial dilution. Specific cDNAs served as quantification standards to determine the absolute transcript copy number for each gene. The gene-specific primer pairs used in the qPCR experiments are detailed in Supplementary Table S3. Absolute quantification of gene expression was conducted through qPCR analysis using the 7300 Real-Time PCR System (Applied Biosystems, Foster City, CA, USA) [29]. The PCR conditions included an initial incubation at 50 °C for 2 min, followed by denaturation at 95 °C for 10 min, and 40 cycles of amplification (95 °C for 15 s and annealing at specific temperatures: 54 °C for *SlPIP2;1*, 52 °C for *SlPIP2;4*, 48 °C for *SlPIP2;6*, 54 °C for *SlPIP2;8*, and 53 °C for *SlPIP2;9* for 1 min). The final steps consisted of 95 °C for 15 s, followed by 60 °C for 30 s, and 95 °C for 15 s. Expression levels of SlPIP2 genes were analyzed using absolute quantification. Each sample was measured in three technical replicates, and the experiment was conducted with three independent biological replicates.

### 2.8. Statistical Analysis

Statistical analyses were carried out using IBM SPSS Statistics version 25. Significant differences were assessed through one-way ANOVA followed by Tukey’s HSD test, with significance set at *p* < 0.05.

## 3. Results

### 3.1. Water Transport Activity

**Figure 1:**
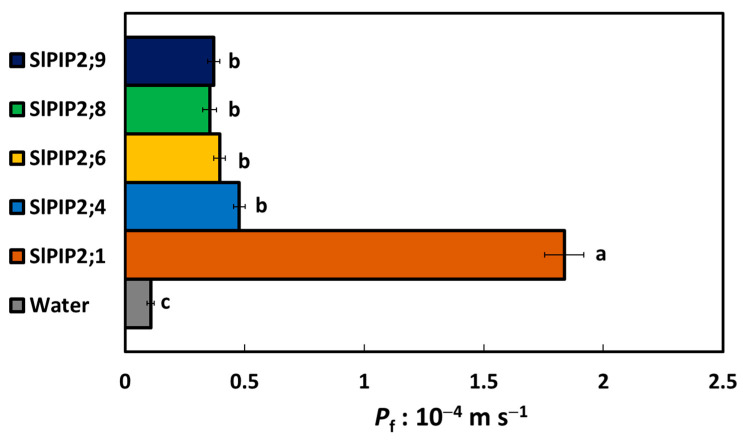
Water transport activity of SlPIP2s. *P*_f_ was determined from swelling assay using *X. laevis* oocytes. Oocytes were injected with 10 ng of *SlPIP2s* cRNA, while those injected with nuclear-free water served as negative controls. Following injection, oocytes were incubated at 18 °C for 24 to 48 h in MBS before the experiments were conducted. All SlPIP2 isoforms (SlPIP2;1, SlPIP2;4, SlPIP2;6, SlPIP2;8, and SlPIP2;9) significantly increased *P*_f_ compared to those injected with water. Statistical significance (*p* < 0.05) was assessed using one-way ANOVA followed by Tukey’s HSD test, with different letters denoting significant differences. Data are means ± SE (*n* = 12–18).

The water transport activity of selected SlPIP2 isoforms was assessed by measuring the osmotic *P*_f_ in *X. laevis* oocytes expressing individual SlPIP2 proteins. As shown in Figure 1, all tested SlPIP2 isoforms (SlPIP2;1, SlPIP2;4, SlPIP2;6, SlPIP2;8, and SlPIP2;9) significantly increased *P*_f_ of oocytes compared to water-injected control ones, confirming their functionality as AQPs. Among these, SlPIP2;1 showed the highest *P*_f_ value, indicating the most significant water transport activity, followed by SlPIP2;4. SlPIP2;6, SlPIP2;8, and SlPIP2;9, which also enhanced *P*_f_ significantly relative to the control, but to a lesser extent compared to SlPIP2;1. These results suggested functional variability among SlPIP2 isoforms, with SlPIP2;1 emerging as the most efficient in facilitating water permeability across the oocyte membrane.

### 3.2. Ion Transport Activity

**Figure 2:**
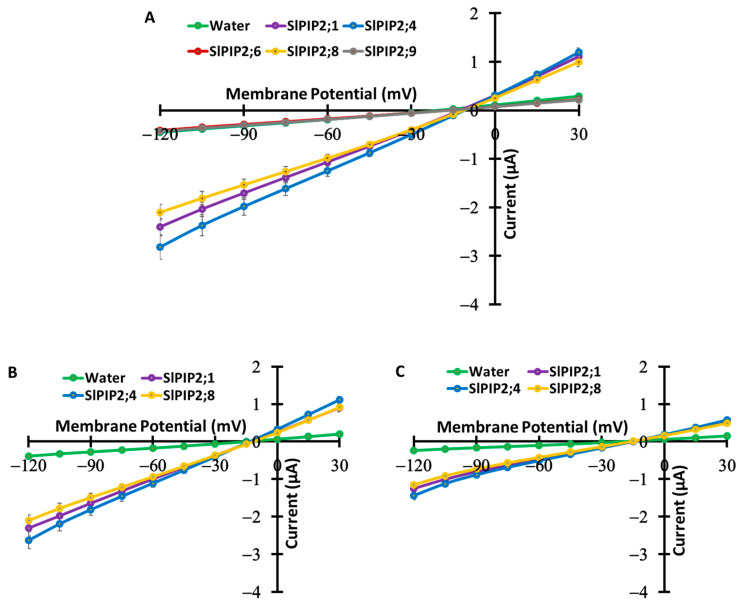
Electrophysiological analysis of SlPIP2 ion transport in *X. laevis* oocytes under different calcium conditions. (**A**–**C**) Current–voltage (I-V) relationships were recorded from oocytes expressing different SlPIP2 isoforms in a solution containing 86.4 mM NaCl and 9.6 mM KCl, with either 30 µM free-Ca^2+^ (**A**,**B**) or 1.8 mM Ca^2+^ (**C**). Each oocyte was injected with 10 ng of *SlPIP2* cRNA or water (control) prior to measurements. A step pulse protocol was applied, ranging from −120 mV to +30 mV with 15 mV increments. The calculation of ionic conductance was based on data recorded at membrane potentials ranging from −75 mV to −120 mV. Among all SlPIP2s, only SlPIP2;1, SlPIP2;4, and SlPIP2;8 showed ionic currents compared to water-injected control. High Ca^2+^ condition (1.8 mM) markedly inhibited ionic currents compared to low Ca^2+^ (30 µM free-Ca^2+^). Data are means ± SE (*n* = 20–27 for (**A**), *n* = 8–9 for (**B**), and *n* = 9–10 for (**C**)).

To assess the ion transport activity of SlPIP2s, TEVC experiments were performed using *X. laevis* oocytes expressing SlPIP2 isoforms (SlPIP2;1, SlPIP2;4, SlPIP2;6, SlPIP2;8, and SlPIP2;9). The results revealed that only SlPIP2;1, SlPIP2;4, and SlPIP2;8 elicited large, bidirectional, and voltage-independent currents (−2.41 ± 0.18 µA, −2.82 ± 0.25 µA, and −2.10 ± 0.16 µA at −120 mV) in a bath solution containing 86.4 mM NaCl, 9.6 mM KCl, and 30 µM free-Ca^2+^ (low Ca^2+^ condition). In contrast, SlPIP2;6 and SlPIP2;9 did not exhibit significant currents (−0.41 ± 0.03 µA and −0.43 ± 0.04 µA at −120 mV) compared to water-injected oocytes (−0.46 ± 0.04 µA at −120 mV) (Figure 2A). Under low Ca^2+^ conditions, SlPIP2;1, SlPIP2;4, and SlPIP2;8 exhibited ionic conductance values of 22.7 µS, 26.8 µS, and 18.7 µS, respectively. In contrast, water-injected oocytes, along with SlPIP2;6 and SlPIP2;9, displayed significantly lower conductance levels of 4.40 µS, 4.00 µS, and 4.10 µS, respectively (Supplementary Table S4). Furthermore, when the external Ca^2+^ concentration increased to 1.8 mM (high Ca^2+^ condition), the currents associated with SlPIP2;1, SlPIP2;4, and SlPIP2;8 were reduced compared to those in low Ca^2+^ conditions (Figure 2B,C). Specifically, the ionic conductance of SlPIP2;1, SlPIP2;4, and SlPIP2;8 was 21.9, 26.1, and 19.8 µS, respectively, under low Ca^2+^ conditions, whereas it decreased to 13.8, 16.8, and 13.0 µS in high Ca^2+^ conditions. These values were significantly higher than those of water-injected oocytes, which exhibited conductance of 3.65 µS in low and 2.37 µS in high Ca^2+^ conditions (Supplementary Table S4).

### 3.3. Cl^−^ Impermeability

**Figure 3:**
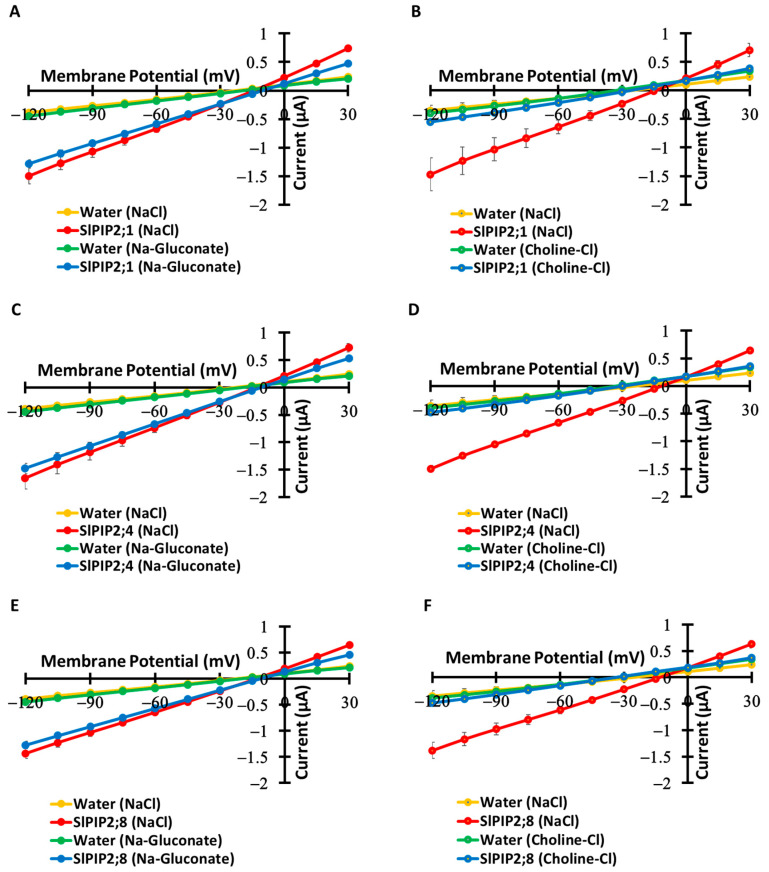
Cl^−^ independence of Na^+^ transport mediated by SlPIP2;1, SlPIP2;4, and SlPIP2;8. (**A**,**C**,**E**) Current–voltage relationships were measured in *X. laevis* oocytes expressing SlPIP2;1, SlPIP2;4, or SlPIP2;8, or injected with water as a control, in the presence of either 96 mM NaCl or 96 mM Na-gluconate. (**B**,**D**,**F**) Current–voltage relationships were similarly recorded under conditions where oocytes were exposed to either 96 mM NaCl or 96 mM Choline-Cl. All experimental solutions contained 30 µM free-Ca^2+^. Oocytes were injected with 10 ng of *SlPIP2;1*, *SlPIP2;4*, or *SlPIP2;8* cRNA. SlPIP2;1, SlPIP2;4, and SlPIP2;8 exhibited Cl^−^-independent Na^+^ transport. Data are means ± SE (*n* = 8–11 for (**A**,**C**,**E**), *n* = 4–7 for (**B**,**D**,**F**)).

To investigate the influence of external anions, specifically Cl^−^, on ion transport through SlPIP2s (SlPIP2;1, SlPIP2;4, and SlPIP2;8), oocytes were exposed to bath solutions containing 96 mM NaCl, 96 mM Na-gluconate, and 96 mM Choline-Cl (Figure 3). Current–voltage recordings showed that the oocytes expressing SlPIP2;1, SlPIP2;4, and SlPIP2;8 responded with similar currents regardless of whether Cl^−^ (−1.49 ± 0.14 µA—Figure 3A, −1.66 ± 0.19 µA—Figure 3C, −1.44 ± 0.09 µA—Figure 3E at −120 mV) or gluconate (−1.28 ± 0.07 µA—Figure 3A, −1.48 ± 0.09 µA—Figure 3C, −1.27 ± 0.07 µA—Figure 3E at −120 mV) was present. The reversal potential remained unchanged across the different solutions, indicating that Cl^−^ does not significantly influence the currents mediated by SlPIP2s. In the presence of 96 mM Choline-Cl, oocytes expressing the SlPIP2;1, SlPIP2;4, and SlPIP2;8 generated only small currents (−0.55 ± 0.04 µA—Figure 3B, −0.48 ± 0.03 µA—Figure 3D, −0.49 ± 0.02 µA—Figure 3F at −120 mV), which were similar to the currents observed in water-injected controls (−0.39 ± 0.05 µA at −120 mV) and notably lower than those seen in the presence of 96 mM NaCl (−1.48 ± 0.29 µA—Figure 3B, −1.49 ± 0.06 µA—Figure 3D, −1.38 ± 0.15 µA—Figure 3F at −120 mV). A shift in the reversal potential was observed among the different solutions, indicating that the Na^+^-induced currents mediated by SlPIP2s are independent of Cl^−^ concentration. Additional experiments, which used solutions containing either 9.6 mM NaCl or 86.4 mM Na-gluconate (giving a total Na^+^ concentration of 96 mM), as well as 9.6 mM NaCl or 86.4 mM Choline-Cl (where the Na^+^ concentration was 9.6 mM), confirmed that a shift in the reversal potential occurred with reduced Na^+^ concentration, as predicted by the Nernst equation. This data further supports the conclusion that Cl^−^ does not influence SlPIP2s-mediated ion currents (Supplementary Figure S2).

### 3.4. Selectivity Sequence of Monovalent Alkaline Cations

**Figure 4:**
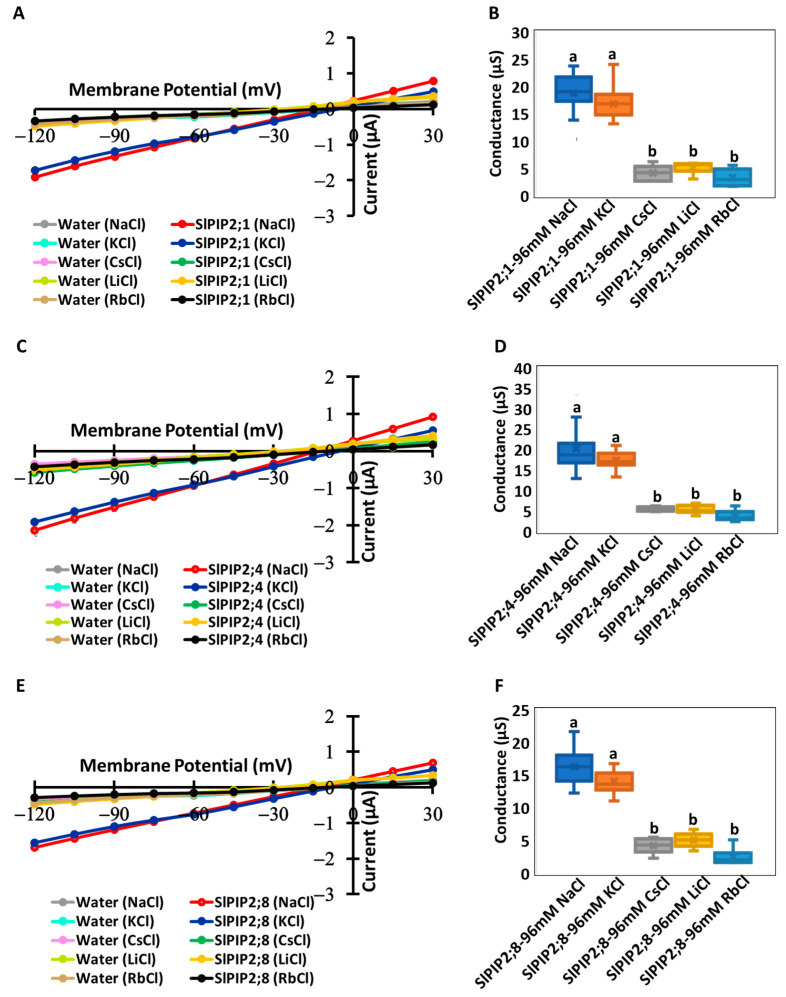
Ion conductance and selectivity of monovalent alkaline cations in SlPIP2;1, SlPIP2;4, and SlPIP2;8. Current–voltage relationships were recorded from *X. laevis* oocytes expressing SlPIP2;1 (**A**), SlPIP2;4 (**C**), SlPIP2;8 (**E**), or injected with water as a control. The results indicate that SlPIP2;1, SlPIP2;4, and SlPIP2;8 exhibit distinct selectivity for monovalent alkaline cations. Oocytes were sequentially exposed to bath solutions containing low calcium (30 µM free-Ca^2+^) and supplemented with 96 mM of Na^+^, K^+^, Cs^+^, Rb^+^, or Li^+^ (as chloride salts). The ionic conductance of SlPIP2;1 (**B**), SlPIP2;4 (**D**), and SlPIP2;8 (**F**) expressing oocytes was measured over a membrane potential range from −75 mV to −120 mV. Each oocyte was injected with 10 ng of *SlPIP2;1*, *SlPIP2;4*, or *SlPIP2;8* cRNA. SlPIP2 isoforms exhibit selective permeability to Na^+^ and K^+^, but no Cs^+^, Li^+^, or Rb^+^ transport. Significant differences (*p* < 0.05) were identified through one-way ANOVA with Tukey’s HSD test and are represented by distinct letters. Data are means ± SE (*n* = 5–15).

Current–voltage relationships were measured for oocytes expressing SlPIP2s (SlPIP2;1, SlPIP2;4, and SlPIP2;8) in the presence of 96 mM Na^+^, K^+^, Cs^+^, Li^+^, or Rb^+^ (as chloride salts; Figure 4). Oocytes in 96 mM solution containing CsCl, LiCl, or RbCl showed no significantly different currents from those of water-injected negative control oocytes. However, currents were detected significantly in SlPIP2s-expressing oocytes when bathed in solutions containing either NaCl or KCl (Figure 4A,C,E). The ionic conductance of SlPIP2;1-, SlPIP2;4-, and SlPIP2;8-expressing oocytes was 18.8, 20.3, and 16.3 µS in 96 mM NaCl solution, respectively, while in a 96 mM KCl solution, it measured 16.8, 17.4, and 14.1 µS (Figure 4B,D,F). These values were significantly higher than those observed in water-injected control oocytes (Supplementary Figure S3). As well as currents, no significant difference in ionic conductance was detected when the oocytes were bathed in 96 mM CsCl (Figure 4B), LiCl (Figure 4D), or RbCl (Figure 4F) compared to water-injected control oocytes (Supplementary Figure S3). These results suggest that SlPIP2;1, SlPIP2;4, and SlPIP2;8 are selectively permeable to Na^+^ and K^+^, but not to Rb^+^, Cs^+^, or Li^+^. The reversal potentials of SlPIP2;1-, SlPIP2;4-, and SlPIP2;8-expressing oocytes were −12.9 mV, −13.5 mV, and −13.4 mV, respectively, in 96 mM NaCl solution (Supplementary Table S5). In 96 mM KCl, the reversal potentials shifted to −5.84 mV, −5.27 mV, and −7.27 mV. In 96 mM CsCl, they were recorded at −13.4 mV, −12.1 mV, and −12.4 mV, whereas in LiCl, the values dropped significantly to −23.5 mV, −24.7 mV, and −24.4 mV. In RbCl solutions, the reversal potentials were −10.6 mV, −9.3 mV, and −9.9 mV. Using these values, ion permeability ratios (P_ion_/P_Na_) were estimated based on the modified Goldman equation (Supplementary Table S5). Among all tested ions, K^+^ numerically exhibited the highest permeability ratio, whereas Li^+^ showed the lowest (Supplementary Table S5).

### 3.5. Effects of Divalent Cations on Ion Transport Activity

**Figure 5:**
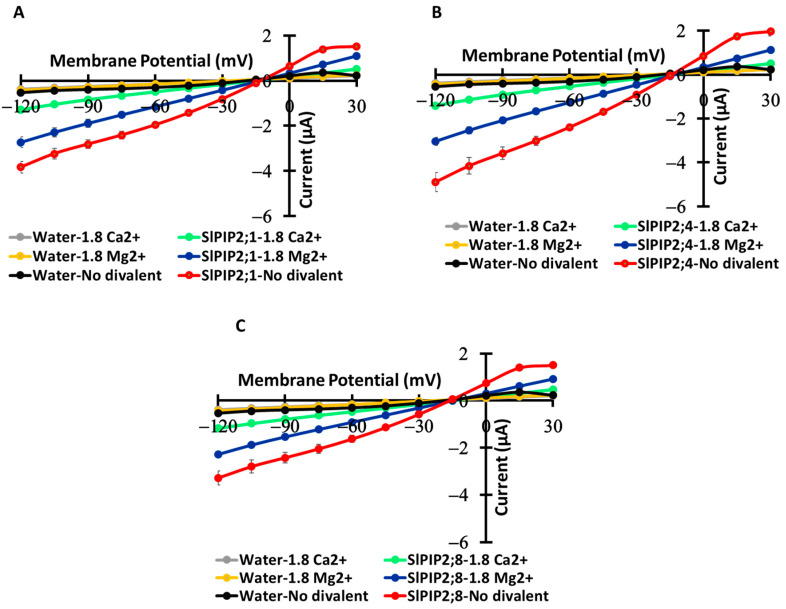
Regulation of ion currents by divalent cations in oocytes expressing SlPIP2;1, SlPIP2;4, and SlPIP2;8. Panels (**A**–**C**) illustrate the effects of divalent cations on ion currents in oocytes expressing SlPIP2;1, SlPIP2;4, and SlPIP2;8. Oocytes were initially bathed in a solution containing 1.8 mM Ca^2+^, which was then replaced with either 1.8 mM Ca^2+^ or 1.8 mM Mg^2+^ (both as chloride salts), along with 86.4 mM NaCl and 9.6 mM KCl. Current–voltage relationships were recorded from *X. laevis* oocytes injected with 10 ng of *SlPIP2* cRNA per oocyte. Water-injected oocytes from the same batch served as negative controls. Ca^2+^ markedly inhibits SlPIP2-mediated currents, while Mg^2+^ has less effect. Data are means ± SE (*n* = 8–13).

SIPIP2;1, SIPIP2;4, and SIPIP2;8 exhibit different ion transport activity in response to divalent cations (Ca^2+^ and Mg^2+^) (Figure 5). In the absence of divalent cations, all three isoforms display higher ion transport. When Ca^2+^ is present (1.8 mM), ion transport is significantly reduced, suggesting an inhibitory effect of Ca^2+^ on these AQPs. Mg^2+^ also decreases ion transport, but to a lesser extent than Ca^2+^. In the presence of 1.8 mM Ca^2+^, oocytes expressing SlPIP2;1, SlPIP2;4, and SlPIP2;8 exhibited relatively small currents of −1.29 ± 0.09 µA (Figure 5A), −1.41 ± 0.05 µA (Figure 5B), and −1.17 ± 0.04 µA (Figure 5C), respectively, at −120 mV. When 1.8 mM Mg^2+^ was replaced with Ca^2+^, the currents increased to −2.73 ± 0.24 µA for SlPIP2;1 (Figure 5A), −3.04 ± 0.18 µA for SlPIP2;4 (Figure 5B), and −2.28 ± 0.09 µA for SlPIP2;8 (Figure 5C) at the same membrane potential. The highest current values were recorded under conditions without divalent cations, reaching −3.84 ± 0.25 µA (Figure 5A), −4.89 ± 0.43 µA (Figure 5B), and −3.29 ± 0.30 µA (Figure 5C) for SlPIP2;1, SlPIP2;4, and SlPIP2;8, respectively, at −120 mV. Water-injected controls show minimal changes, confirming that the observed effects are specific to SIPIP2 isoforms (Figure 5). These results suggest that Ca^2+^ and Mg^2+^ regulate ion permeability through SIPIP2s, with Ca^2+^ exerting a more inhibitory influence.

### 3.6. Na^+^ Concentration-Dependent Ionic Conductance

**Figure 6:**
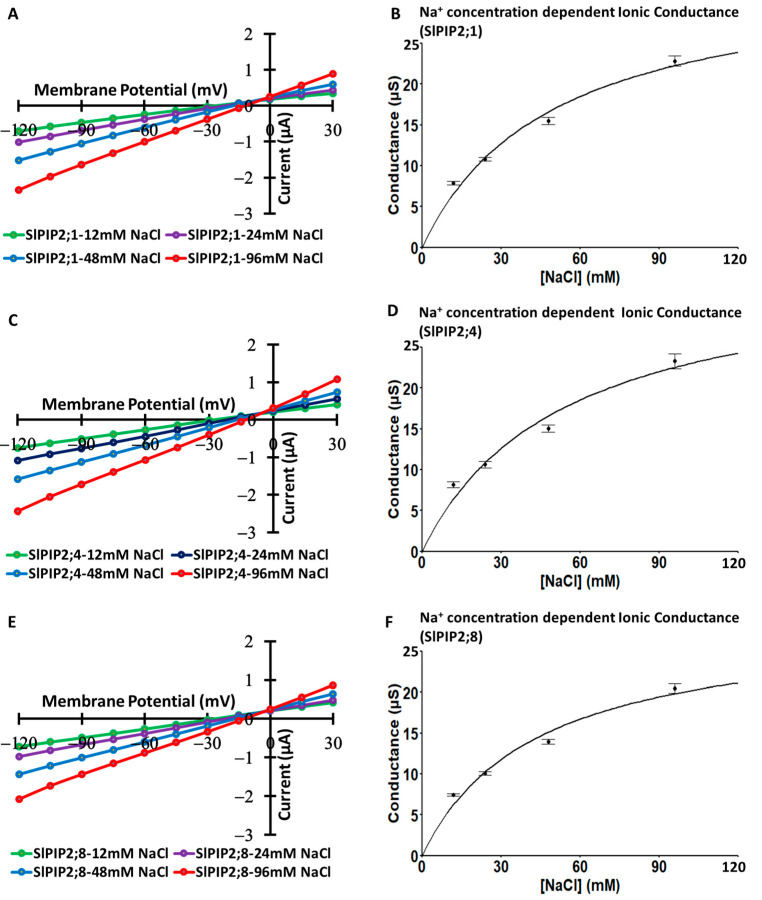
Analysis of icAQP activity in SlPIP2;1, SlPIP2;4, and SlPIP2;8 under different Na^+^ concentrations. TEVC experiments were conducted using *X. laevis* oocytes. (**A**,**C**,**E**) Ion current responses were measured in oocytes expressing SlPIP2;1, SlPIP2;4, and SlPIP2;8 in the presence of 12, 24, 48, or 96 mM NaCl with 30 µM free-Ca^2+^ (*n* = 10–12). A step pulse protocol was applied, ranging from −120 mV to +30 mV with 15 mV increments for each oocyte. Enzyme kinetic analysis was performed on conductance data (µS) recorded at membrane potentials between −75 and −120 mV, derived from the experiments shown in panels (**B**,**D**,**F**). SlPIP2;1, SlPIP2;4, and SlPIP2;8 function as very low-affinity Na^+^ transporters.

The ionic conductance of SlPIP2s (SlPIP2;1, SlPIP2;4, and SlPIP2;8) was further analyzed in rising NaCl concentrations from 12 mM to 96 mM (Supplementary Table S6) and followed by Michaelis–Menten curve fitting (Figure 6). The apparent maximum conductance (*V*max) and apparent Michaelis constant (*K*m) were 25.6 µS and 28.2 mM, respectively, in SlPIP2;1 (Figure 6B), SlPIP2;4 showing 24.4 µS and 25.3 mM (Figure 6D), and SlPIP2;8 displaying 22.3 µS and 24.9 mM (Figure 6F).

### 3.7. K^+^ Concentration-Dependent Ionic Conductance

**Figure 7:**
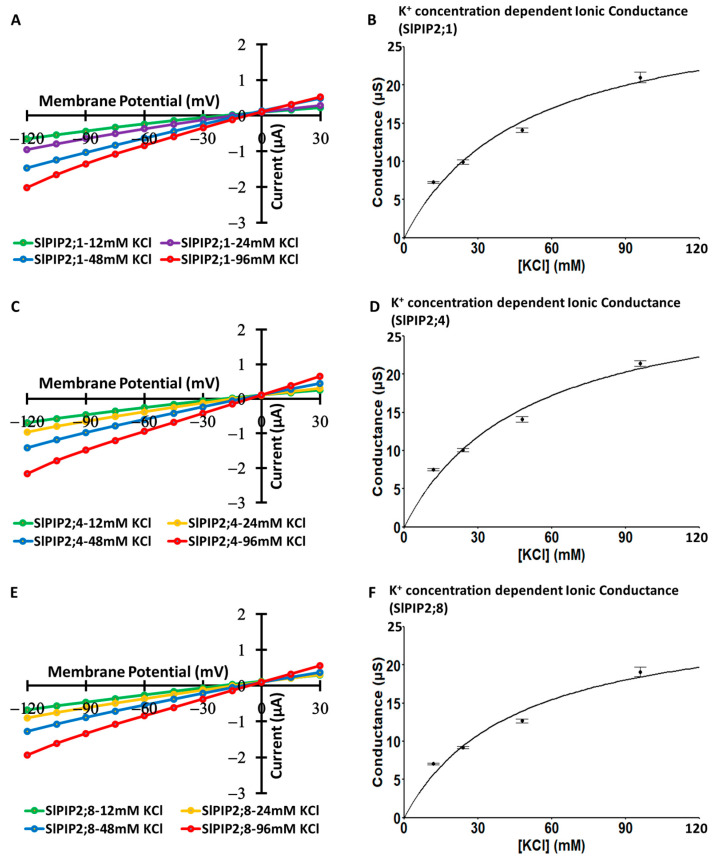
Assessment of icAQP activity in SlPIP2;1, SlPIP2;4, and SlPIP2;8 under different K^+^ concentrations. TEVC experiments were carried out using *X. laevis* oocytes. (**A**,**C**,**E**) Ion current responses were measured in oocytes expressing SlPIP2;1, SlPIP2;4, and SlPIP2;8 exposed to 12, 24, 48, or 96 mM KCl with 30 µM free-Ca^2+^ (*n* = 10–12). A step pulse protocol was applied, ranging from −120 mV to +30 mV with 15 mV increments. Conductance data (µS) recorded at membrane potentials between −75 and −120 mV were analyzed using enzyme kinetics, based on the experiments shown in panels (**B**,**D**,**F**). SlPIP2;1, SlPIP2;4, and SlPIP2;8 function as very low-affinity K^+^ transporters.

The ionic conductance of SlPIP2;1, SlPIP2;4, and SlPIP2;8 was analyzed in rising KCl, like NaCl, with concentrations ranging from 12 mM to 96 mM (Supplementary Table S7) and followed by Michaelis–Menten curve fitting (Figure 7). The apparent *V*max and apparent *K*m were determined as follows: 23.3 µS and 27.6 mM for SlPIP2;1 (Figure 7B), 22.9 µS and 25.9 mM for SlPIP2;4 (Figure 7D), and 20.0 µS and 23.3 mM for SlPIP2;8 (Figure 7F).

### 3.8. Expression of SlPIP2s in Tomato Plants

**Figure 8:**
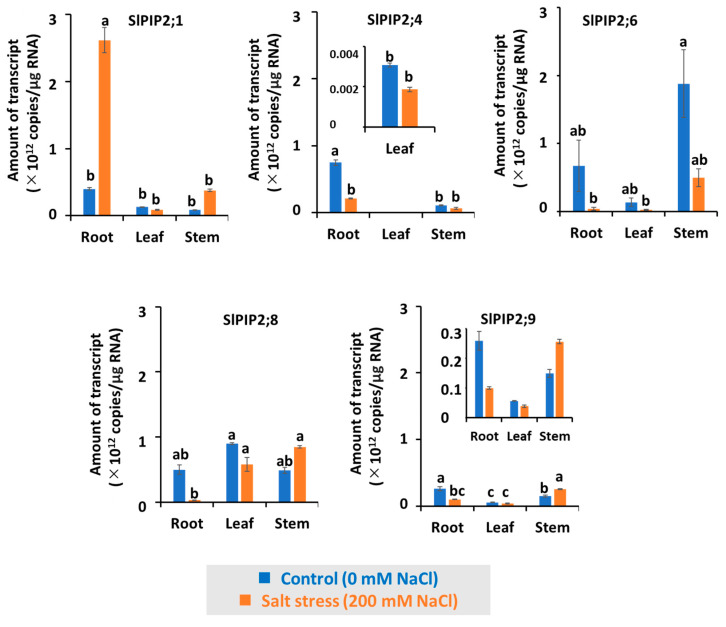
Quantification of *SlPIP2*s transcript levels in the Micro-Tom tomato cultivar using qPCR. Plants were grown in soil-filled pots for approximately 30 days before being divided into two treatment groups: three plants were irrigated with 200 mM NaCl in tap water, while another three received only tap water for 17 days. The transcript levels of *SlPIP2*s were measured in leaves, stems, and roots through absolute quantification, expressed as copies per microgram of RNA. Among the icAQPs, *SlPIP2;4* and *SlPIP2;8* show relatively low and stable expression levels, whereas the amount of *SlPIP2;1* transcript increased significantly in roots after 17 days of 200 mM NaCl treatment. Significant differences (*p* < 0.05) were determined using one-way ANOVA followed by Tukey’s HSD test and are indicated by different letters. Data are means ± SE (*n* = 3).

This study examined the expression levels of *SlPIP2* transcripts in the leaves, stems, and roots of Micro-Tom tomato plants under both control and salt stress conditions (Figure 8). Using qPCR with absolute quantification, we assessed the impact of 200 mM NaCl treatment over 17 days. Expression of *SlPIP2* was significantly altered in response to salt stress. In the roots, all *SlPIP2s* (*SlPIP2;4*, *SlPIP2;6*, *SlPIP2;8*, and *SlPIP2;9*) exhibited higher transcript abundance under control conditions compared to saline treatment, except for *SlPIP2;1*, which showed a significant increase in expression after 17 days of salt exposure. In the leaves, transcript levels of all *SlPIP2s* remained relatively stable between control and salt-treated samples, with only a slight increase in *SlPIP2;6* expression under control conditions. In the stems, the expression of *SlPIP2;1* and *SlPIP2;4* remained unchanged between treatments. However, *SlPIP2;6* exhibited the highest transcript abundance under control conditions, while *SlPIP2;8* and *SlPIP2;9* also showed slightly increased expression in response to salt stress.

## 4. Discussion

Salinity stress is a major abiotic factor limiting agricultural productivity, particularly in arid and semi-arid regions [30]. It disrupts germination, growth, photosynthesis, and stomatal behavior while inducing osmotic and ionic stress through reduced water availability, ROS accumulation, ion toxicity, and nutrient imbalance [31]. AQPs, especially the PIP subfamily, one of the largest groups within the AQP superfamily, are localized to the plasma membrane where their members facilitate the transport of water and small neutral solutes, playing a crucial role in maintaining water homeostasis and conferring tolerance to diverse abiotic stresses, including salinity [32,33]. Thus, PIPs are key to plant resilience under salinity and offer promising targets for breeding salt-tolerant crops.

Five SlPIP2s were selected to identify icAQPs. This selection was based on the fact that among the remaining SlPIP2s, SlPIP2;2, SlPIP2;3, and SlPIP2;7 are not considered true AQPs; SlPIP2;5 is not expressed in any tissue; SlPIP2;11 lacks EST (expressed sequence tag) evidence; and SlPIP2;12 is a pseudogene. Furthermore, we focused exclusively on PIP2 isoforms because PIP1 proteins are generally not functional in the oocyte membrane, whereas PIP2 proteins are known to be functional in the *X. laevis* oocyte system used for TEVC experiments.

In the present study, *X. laevis* oocytes expressing SlPIP2 isoforms showed significantly higher *P*_f_ than the controls. This indicates that all SlPIP2 proteins are properly localized and functional at the plasma membrane of the oocytes. Among all SlPIP2 isoforms, SlPIP2;1 exhibited the highest activity (Figure 1). The high-water permeability observed in SlPIP2;1 may be attributed to its hypothetically wider monomeric pore compared to other isoforms. A similarly wide monomeric pore was previously reported for BraPIP2;1 [34]. This indicates functional differences among SlPIP2s in aquaporin’s water transport activity, localization, or regulation. Such differences may play a key role in plant water homeostasis and stress adaptation. Previous studies also highlight AQPs as membrane proteins that facilitate water transport and are tightly regulated under water deficit [28,35]. In tobacco, NtAQP1 may play a unique role in absorbing residual water during drought stress, thereby helping to protect the plant from drought [29]. Further research could enhance understanding of their regulations to improve water use efficiency in crops.

SlPIP2;1, SlPIP2;4, and SlPIP2;8 exhibited strong, voltage-independent bidirectional ion currents, whereas SlPIP2;6 and SlPIP2;9 lacked such activity (Figure 2A). What structural or amino acid mutations could cause these differences? We have initiated studies to determine if amino acids are responsible for the ion transport. These investigations will help elucidate the molecular basis that distinguishes icAQPs from non-icAQPs. Moreover, we aim to generate a knockout line of transgenic tomato icAQPs. In parallel, we will create two artificial mutants for each tomato icAQP: one that retains only ion transport activity without water transport, and another that retains only water transport activity without ion transport. These artificial mutants, along with the wild type, will then be introduced into the knockout line to confirm the physiological roles of tomato icAQPs in salt stress. Notably, inward currents recorded for SlPIP2;1, SlPIP2;4, and SlPIP2;8 under low external Ca^2+^ conditions (30 µM free-Ca^2+^) decreased significantly under high Ca^2+^ conditions (1.8 mM), representing an approximate 45% inhibition (Figure 2B,C). This calcium-dependent suppression may occur either through direct interaction with the channel or via Ca^2+^-activated signaling mechanisms. A similar inhibitory pattern was previously reported for the ion-transporting icAQPs, AtPIP2;1 [6], HvPIP2;8 [10], and OsPIP2;4 [11]. Given the well-established role of Ca^2+^ in mitigating Na^+^ toxicity [36], recent studies showed that Ca^2+^ reduces the ionic currents of icAQPs [2]. This Ca^2+^-dependent current reduction through icAQPs may represent an important initial step in mitigating salt stress, particularly in the context of molecular breeding targeting icAQPs. These findings suggest that SlPIP2;1, SlPIP2;4, and SlPIP2;8 may play a role in calcium-modulated water and ion homeostasis in tomato plants.

Electrophysiological analyses revealed that SlPIP2;1, SlPIP2;4, and SlPIP2;8 selectively conduct Na^+^ while excluding Cl^−^, indicating strong cation selectivity (Figure 3). Comparable current amplitudes in NaCl and Na-gluconate solutions suggest that Cl^−^ does not contribute substantially to ion flux (Figure 3A,C,E). Substitution of Na^+^ with choline in Choline-Cl solution led to a pronounced decrease in current, confirming Na^+^ as the primary charge carrier (Figure 3B,D,F). Moreover, a shift in reversal potential, upon reducing Na^+^ concentration from 96 mM to 9.6 mM, corresponded with the Nernst prediction, reinforcing Na^+^-dependent conductance (Supplementary Figure S2). The exclusion of Cl^−^ is likely due to the electrostatic environment within the SlPIP2 pore, which disfavors anion permeation, as well as Cl^−^’s larger hydration shell and higher desolvation energy. These results are consistent with prior findings in plant AQPs and cation-selective channels [10,11], supporting the classification of SlPIP2s as cation-conducting AQPs with high ion selectivity. In contrast, cation transport mediated by AtPIP2;1 was previously reported to be Cl^−^-dependent [6].

SlPIP2 isoforms exhibit selective permeability to Na^+^ and K^+^, while largely excluding Cs^+^, Li^+^, and Rb^+^, as shown by robust currents in Na^+^ and K^+^ solutions and minimal conductance in the presence of other monovalent cations (Figure 4). These results strongly suggest that SlPIP2s preferentially transport Na^+^ and K^+^, indicating NSCC activity for Na^+^ and K^+^. A similar NSCC activity was previously reported for the ion-transporting icAQPs, AtPIP2;1 [6], HvPIP2;8 [10], and OsPIP2;4 [11]. An analysis of ion permeability ratios further suggested a selectivity sequence of SlPIP2s: K^+^ ≈ Na^+^ (Supplementary Table S5). Although reversal potential analyses indicated numerically greater permeability for K^+^, the conductance was numerically higher in Na^+^ solutions due to the higher diffusion coefficient of Na^+^, which allows it to pass through the pore more rapidly than K^+^. This selectivity likely arises from structural features that favor smaller, biologically relevant cations, while excluding larger ions such as Cs^+^ and Rb^+^, as well as those with higher hydration energies, like Li^+^. In contrast, the currents in CsCl, LiCl, and RbCl solutions did not differ significantly from the water controls, indicating that their reversal potentials and ion permeability ratios are unlikely to have any physiological relevance (Supplementary Table S5). The ability of SlPIP2s to facilitate Na^+^ and K^+^ transport while excluding non-essential cations suggests a critical role in maintaining ion homeostasis under salt stress, where precise Na^+^ and K^+^ balance is vital for cellular function and osmotic regulation [37]. Similar regulation of Na^+^ transport in response to external K^+^ levels was reported in HKT-type transporters such as wheat TaHKT1;5-D and wheat TmHKT1;5-A, which reduce Na^+^ uptake under high K^+^ conditions [38].

The regulation of SlPIP2 isoforms (SlPIP2;1, SlPIP2;4, and SlPIP2;8) by divalent cations underscores an important mechanism for fine-tuning ion permeability. Experimental data show that Ca^2+^ markedly reduces SlPIP2-mediated currents, with Mg^2+^ having a weaker but still noticeable effect (Figure 5). This inhibition likely occurs through multiple mechanisms, including direct pore blockage, electrostatic interactions, and structural modifications. Similar regulatory patterns are seen in *Arabidopsis* AQPs AtPIP2;1 and AtPIP2;2, which conduct Na^+^ and are highly sensitive to Ca^2+^, Mg^2+^, Ba^2+^, and Cd^2+^ [6,9]. Comparable sensitivity is reported in barley HvPIP2;8 and rice OsPIP2;4, particularly to Ca^2+^, Ba^2+^, and Cd^2+^, while Mg^2+^ exerts a weaker effect [10,11]. The relatively mild impact of Mg^2+^ suggests lower binding affinity or transient interactions compared to Ca^2+^, reinforcing Ca^2+^’s role as a key regulator of PIP2-mediated ion transport.

Ionic conductance analysis of SlPIP2;1, SlPIP2;4, and SlPIP2;8 shows increased conductance with rising NaCl concentrations (12–96 mM), confirming their dependence on extracellular Na^+^ and their role in Na^+^ transport (Supplementary Table S6). Peak conductance at 96 mM NaCl reached 22.8 µS (SlPIP2;1), 23.3 µS (SlPIP2;4), and 20.4 µS (SlPIP2;8). Apparent Michaelis–Menten kinetics revealed the highest *V*max in SlPIP2;1 (25.6 µS), followed by SlPIP2;4 (24.4 µS) and SlPIP2;8 (22.3 µS), with respective *K*m values of 28.2 mM, 25.3 mM, and 24.9 mM (Figure 6B,D,F). These parameters identify SlPIP2s as low-affinity Na^+^ transporters, contrasting with the high-affinity Na^+^ transporters, such as HKT1 proteins [39]. Their Na^+^ conductance likely supports ion influx under saline conditions. Similarly, OsPIP2;4 also functions as a low-affinity Na^+^ channel in the root surface region and may serve as a major entry point for Na^+^ influx from saline soil into plants [11]. However, OsPIP2;4 has been proposed to mediate Na^+^ exclusion by facilitating Na^+^ uptake into endodermal and pericycle cells, similar to OsHKT1;5 [40], which contributes to salt tolerance [11].

Similarly, SlPIP2;1, SlPIP2;4, and SlPIP2;8 exhibit increased conductance with rising KCl concentrations, reaching peak values of 20.9 µS, 21.4 µS, and 19.1 µS, respectively, at 96 mM KCl (Supplementary Table S7), indicating their role in K^+^ transport. Michaelis–Menten analysis showed that SlPIP2;1 has the highest transport capacity (23.3 µS), followed by SlPIP2;4 (22.9 µS) and SlPIP2;8 (20.0 µS), with *K*m values of 27.6, 25.9, and 23.3 mM, respectively (Figure 7B,D,F). These kinetic profiles classify them as low-affinity K^+^ transporters when compared with high-affinity K^+^ transporters such as HAK5 proteins [39]. Their ability to mediate substantial K^+^ flux may support ionic homeostasis under salinity stress. While high-affinity K^+^ uptake systems are crucial in K^+^-deficient soils [41], these SlPIP2 isoforms, being low-affinity K^+^ transporters like OsPIP2;4 [11], warrant further investigation into their potential role in K^+^ acquisition in tomato plants. A similar role has been proposed for OsPIP2;4 in rice, highlighting the need to evaluate its function in conjunction with selective K^+^ transporters to clarify its contribution to overall K^+^ nutrition [11]. Although the low-affinity K^+^ uptake system may be involved in luxury K^+^ absorption, luxury K^+^ absorption has been rarely studied, except in the case of potatoes [42].

Expression analysis of *SlPIP2* transcripts in Micro-Tom tomato plants under control and salt stress conditions revealed distinct tissue-specific responses (Figure 8). In roots, most isoforms (*SlPIP2;4*, *SlPIP2;6*, *SlPIP2;8*, and *SlPIP2;9*) were downregulated under salt stress, consistent with similar findings in barley [28]. However, *SlPIP2;1* showed increased expression after 17 days of treatment, which may be due to its higher water permeability and its potential role in sequestering excess Na^+^ into the vacuole or exporting it from the cytosol through cooperative NHX antiporters or SOS1. This suggests that *SlPIP2;1* contributes to stress adaptation by facilitating the selective transport of water and ions under salt stress conditions. Similar stress-induced upregulation of PIP genes has been reported in cucumber [43] and in tomato under salt stress [44]. In leaves, *SlPIP2* expression remained essentially unchanged, indicating a limited transcriptional response to salinity, with *SlPIP2;6* showing a slight increase under control conditions. This suggests that leaf *SlPIP2*s may contribute more to general water homeostasis than to salt stress responses. In stems, while *SlPIP2;1* and *SlPIP2;4* were stable, *SlPIP2;6* was most abundant under control conditions, and *SlPIP2;8* and *SlPIP2;9* were slightly upregulated under salt stress, implying potential roles in water transport or ion homeostasis. These findings suggest to a coordinated and isoform-specific regulation of SlPIP2 genes in maintaining water and ion balance under salinity. The distinct expression patterns across tissues support the notion that individual *SlPIP2* isoforms have specialized physiological functions, consistent with previous findings that PIP isoforms perform diverse roles [45]. AQPs are also well known for supporting cell expansion and osmotic regulation under stress conditions [46,47]. Notably, this is the first study to demonstrate ion-channel activity in tomato AQPs, suggesting a dual role in water and salinity tolerance. Further functional studies could clarify the contributions of individual SlPIP2 isoforms to salt tolerance in tomato.

## 5. Conclusions

In conclusion, these results demonstrate that among all SlPIP2 isoforms, only SlPIP2;1, SlPIP2;4, and SlPIP2;8 exhibit transport activity for Na^+^ and K^+^, but not Cl^−^. These isoforms act as very low-affinity Na^+^ and K^+^ transporters. Ca^2+^ considerably inhibited these transport activities, whereas Mg^2+^ had a milder impact. Notably, although *SlPIP2;4* and *SlPIP2;8* maintain consistently low expression, *SlPIP2;1* expression is markedly elevated in roots following 17 days of 200 mM NaCl stress, highlighting their distinct physiological roles as icAQPs. Further studies are warranted to elucidate the molecular mechanisms underlying their function as icAQPs under salt stress. These icAQPs should be necessary for plant resilience under salt stress, and there is a possibility to develop salt-tolerant crops with icAQPs.

## Data Availability

The data that support the findings of this study are available from the corresponding author upon reasonable request.

## Supplementary Materials

The following supporting information can be downloaded at https://www.mdpi.com/article/10.3390/cells14171305/s1, Table S1: Primers and PCR conditions used to amplify the full-length PIP2 coding sequence; Table S2: Primers used for subcloning; Table S3: Gene-specific primer pairs used in qPCR experiment; Table S4: Ionic conductance of oocytes injected with SlPIP2s or water in a solution containing 86.4 mM NaCl and 9.6 mM KCl; Table S5: Reversal potential and ion permeability ratios of SlPIP2s in *X. laevis* oocytes; Table S6: Ionic conductance of oocytes injected with SlPIP2s in a solution containing 96, 48, 24, and 12 mM NaCl; Table S7: Ionic conductance of oocytes injected with SlPIP2s in a solution containing 96, 48, 24, and 12 mM KCl; Figure S1. Fresh weight (g) of tomato plant parts (cv. Micro-Tom) in control and salt stressed condition; Figure S2. Na^+^ transport mediated by SlPIP2;1, SlPIP2;4, and SlPIP2;8 is independent of Cl^−^; Figure S3. Monovalent cation conductance in water-injected oocytes.

## Abbreviations

The following abbreviations are used in this manuscript:

AQP	Aquaporin
cRNA	Capped RNA
*P*_f_	Water permeability coefficient
icAQP	Ion-conducting aquaporin
PCR	Polymerase chain reaction
PIP	Plasma membrane intrinsic protein
qPCR	Quantitative PCR
TEVC	Two-electrode voltage clamp
NSCC	Non-selective cation channel
