# Supplementary material for: Discovery and Functional Characterization of Novel Aquaporins in Tomato (Solanum lycopersicum): Implications for Ion Transport and Salinity Tolerance
Source: Cells. 2025 Aug 22;14(17):1305. doi: 10.3390/cells14171305 (PMC12427852; doi:10.3390/cells14171305)
Supplement: Supplementary file 1 [file cells-14-01305-s001.zip › cells-3816163-supplementary.pdf]

Table S1. Primers and PCR conditions used to amplify the full-length PIP2 coding sequence.

Gene	Stretching direction	Primer sequence
SlPIP2;1	FWD	5'- CCTATTCTTTATCTTTCTTTTTGGTG -3'
	REV	5'- GCAAATCTAAAATTAAGTGCCAAA -3'
	94°C 3 min, {94°C 15s, 56°C 20s, 72°C 1min} 35 cycles	
SlPIP2;4	FWD	5'- GGAAAGATGACTAAAGAAGTAACAGA -3'
	REV	5'- TGAGTTGAAACTATTGATCGAA -3'
	94°C 3 min, {94°C 15s, 56°C 20s, 72°C 1min} 35 cycles	
SlPIP2;6	FWD	5'- ATGACGAAAGAAGTCGAAGCGGCTCACG -3'
	REV	5'- TCACGCATTGCTTCTGAATGAAC -3'
	94°C 3 min, {94°C 15s, 62°C 20s, 72°C 1min} 35 cycles	
SlPIP2;8	FWD	5'- TCCGAAGCAAAAATGTCAAA -3'
	FWD	5'- GATTTAATTGGTGGCGTTGC -3'
	94°C 3 min, {94°C 15s, 62°C 20s, 72°C 1min} 35 cycles	
SlPIP2;9	FWD	5'- GCACAAATTCATCATCTTCTTCA -3'
	REV	5'- CAAAATCAAACAATTAAGCCAGAA -3'
	94°C 3 min, {94°C 15s, 61°C 20s, 72°C 1min} 35 cycles	

Table S2. Primers used for subcloning.

Insert gene		Primer sequence
SlPIP2;1	I	5'- GGCAGATCTaccATGGCAAAAGATATGGAGGTTGGTAC -3'
	II	5'- GGTAACCagatctTTAAGCATTGCTCCTGAATGAACCAAG -3'
	III	5'- GAGCAATGCTTAAagatctGGTTACCACTAAACCAGC -3'
	IV	5'- TATCTTTTGCCATggtAGATCTGCCAAAGTTGAGCG -3'
SlPIP2;4	I	5'- GGCAGATCTaccATGACTAAAGAAGTAACAGATTTTTCAGCAAA -3'
	II	5'- GTAACCagatctTCAGGCAGTGCTCCTGAATGAAC -3'
	III	5'- GCACTGCCTGAagatctGGTTACCACTAAACCAGC -3'
	IV	5'- CTTCTTTAGTCATggtAGATCTGCCAAAGTTGAGCG -3'
SlPIP2;6	I	5'- GCAGATCTaccATGACGAAAGAAGTCGAAGCGGC -3'
	II	5'- GTAACCagatctTCACGCATTGCTTCTGAATGAACC -3'
	III	5'- AGCAATGCGTGAagatctGGTTACCACTAAACCAGC -3'
	IV	5'- CTTCTTTCGTCATggtAGATCTGCCAAAGTTGAGCG -3'
SlPIP2;8	I	5'- GGCAGATCTaccATGTCAAAAGAAGTGATTGAAGAAGGAC -3'
	II	5'- GTAACCagatctTTAATTGGTGGCGTTGCTGCGG -3'
	III	5'- CGCCACCAATTAAagatctGGTTACCACTAAACCAGC -3'
	IV	5'- CTTCTTTTGACATggtAGATCTGCCAAAGTTGAGCG -3'
SlPIP2;9	I	5'- GCAGATCTaccATGTCGAAGGACGTGATTGAAGAAG -3'
	II	5'- GGTAACCagatctTTAGTTGGTTTGGTTGCTGCGGAA -3'
	III	5'- CCAAACCAACTAAagatctGGTTACCACTAAACCAGC -3'
	IV	5'- GTCCTTCGACATggtAGATCTGCCAAAGTTGAGCG -3'

The lowercase letters acc indicate the Kozak sequence, and agatct indicates the BglII recognition sequence.

Table S3. Gene-specific primer pairs used in qPCR experiment.

Gene name	Forward primer (5′-3′)	Reverse primer (5′-3′)
SlPIP2;1	GTGCTGCTGTTGTTTATGGACA	CATCCAACACAACTCTAACAAC
SlPIP2;4	CAATGGTGACAAGGCGTGG	GAAGGCGAATTCATAGGAT
SlPIP2;6	CAGAGCATCCTCTGTTT	CACCGCAAATATCGCCTC
SlPIP2;8	GGAGCTGCTGTTATTGCTGA	GCACAGATCCAAGGCTAAGA
SlPIP2;9	GCAATGGCAGCAGCAATATACCA	CGAAAGAGAATAGACCACCA

Table S4. Ionic conductance of oocytes injected with SlPIP2s or water in a solution containing 86.4 mM NaCl and 9.6 mM KCl. Conductance values were determined from membrane potential measurements ranging from −75 mV to −120 mV, as shown in Figure 1. Data are presented as mean ± SE, with sample sizes of n = 20–27 (A), n = 8–9 (B), and n = 9–10 (C). Statistical significance was assessed using one-way ANOVA followed by Duncan's multiple comparisons test, where * indicates $P < 0.05$ and "ns" denotes no significant difference.

Ionic Conductance (μS)	Water (control)	SlPIP2;1	SlPIP2;4	SlPIP2;6	SlPIP2;8	SlPIP2;9
A) Low Ca^{2+} (30 μM)	4.40 ± 0.33^{ns}	$22.7 \pm 1.59^{*}$	$26.8 \pm 2.43^{*}$	4.00 ± 0.30^{ns}	$18.7 \pm 1.30^{*}$	4.10 ± 0.42^{ns}

Ionic Conductance (μS)	Water (control)	SlPIP2;1	SlPIP2;4	SlPIP2;8
B) Low Ca^{2+} (30 μM)	3.65 ± 0.38^{ns}	$21.9 \pm 2.20^{*}$	$26.1 \pm 2.39^{*}$	$19.8 \pm 1.55^{*}$
C) High Ca^{2+} (1.8 mM)	2.37 ± 0.21^{ns}	$13.8 \pm 1.51^{*}$	$16.8 \pm 1.55^{*}$	$13.0 \pm 0.63^{*}$

Table S5. Reversal potential and ion permeability ratios of SlPIP2s in *X. laevis* oocytes. Ion permeability ratios were estimated from shifts in reversal potential recorded in a solution containing 96 mM XCl, where X represents Na, K, Rb, Cs, or Li. The calculations were based on a modified Goldman equation, assuming Cl^- permeability to be negligible. The details of the gray-colored column are discussed in the discussion section.

Solutions / iCAQP	96mM NaCl		96mM KCl		96mM CsCl		96mM LiCl		96mM RbCl	
	Erev	SE(±)	Erev	SE(±)	Erev	SE(±)	Erev	SE(±)	Erev	SE(±)
SlPIP2;1	-12.9	0.52	-5.84	0.33	-13.4	0.62	-23.5	1.31	-10.6	0.82
SlPIP2;4	-13.5	0.32	-5.27	0.25	-12.1	0.26	-24.7	0.26	-9.3	0.64
SlPIP2;8	-13.4	0.36	-7.27	0.24	-12.4	0.82	-24.4	0.49	-9.9	0.37

	Ion	Na^+	K^+	Cs^+	Li^+	Rb^+
SlPIP2;1	Pion/PNa	1	1.33	0.98	0.66	1.10
SlPIP2;4	Pion/PNa	1	1.38	1.05	0.64	1.18
SlPIP2;8	Pion/PNa	1	1.27	1.04	0.64	1.14

Table S6. Ionic conductance of oocytes injected with SlPIP2s in a solution containing 96, 48, 24, and 12 mM NaCl. Conductance values were determined from membrane potential measurements ranging from −75 mV to −120 mV, as shown in Figure 6. Data are presented as mean ± SE, with sample sizes of n = 10–12.

Ionic Conductance (μS)	SlPIP2;1	SlPIP2;4	SlPIP2;8
96 mM NaCl (Low Ca^{2+} -30 μM)	22.8±0.60	23.3±0.89	20.4±0.58
48 mM NaCl (Low Ca^{2+} -30 μM)	15.5±0.43	15.0±0.42	13.9±0.29
24 mM NaCl (Low Ca^{2+} -30 μM)	10.8±0.21	10.6±0.41	10.1±0.23
12 mM NaCl (Low Ca^{2+} -30 μM)	7.9±0.23	8.2±0.37	7.5±0.15

Table S7. Ionic conductance of oocytes injected with SlPIP2s in a solution containing 96, 48, 24, and 12 mM KCl. Conductance values were determined from membrane potential measurements ranging from −75 mV to −120 mV, as shown in Figure 7. Data are presented as mean ± SE, with sample sizes of *n* = 10–12.

Ionic Conductance (μS)	SlPIP2;1	SlPIP2;4	SlPIP2;8
96 mM KCl (Low Ca^{2+} -30 μM)	20.9±0.67	21.4±0.34	19.1±0.62
48 mM KCl (Low Ca^{2+} -30 μM)	14.1±0.31	14.1±0.37	12.7±0.25
24 mM KCl (Low Ca^{2+} -30 μM)	9.9±0.31	10.1±0.21	9.2±0.15
12 mM KCl (Low Ca^{2+} -30 μM)	7.3±0.13	7.5±0.15	7.0±0.12

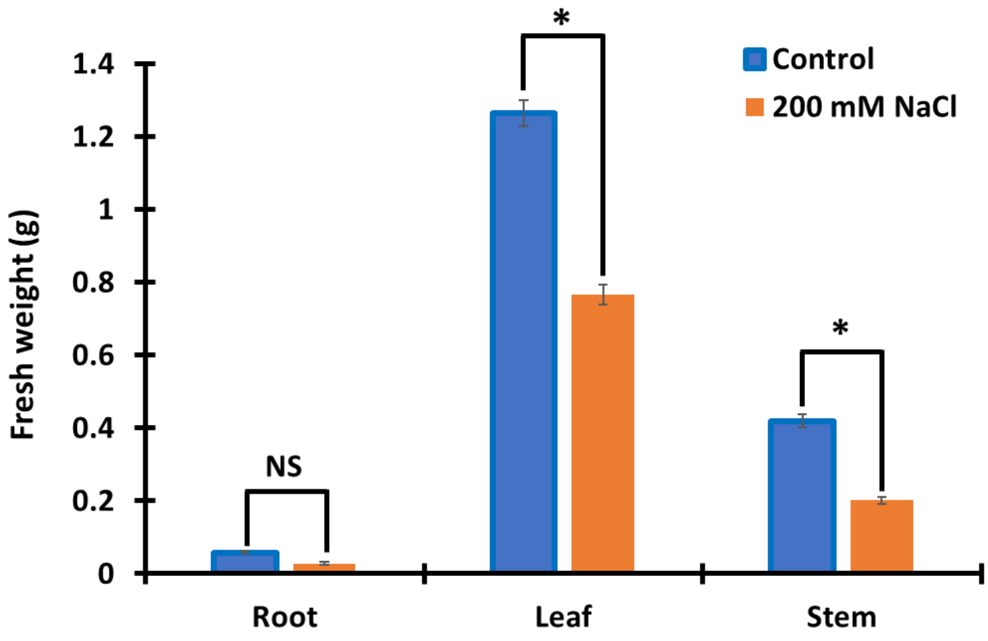

Figure S1. Fresh weight (g) of tomato plant parts (cv. Micro-Tom) in control and salt-stressed conditions. After growing tomato plants in soil-filled pots for roughly 30 days, they were divided into two groups: one irrigated with 200 mM NaCl solution and the other with tap water for 17 days. At approximately 47 days old, leaf, root, and stem samples were collected and analyzed. A significant difference was observed between control and salt-stressed samples in the leaf and stem, but not in the root samples. Significant differences ($p < 0.05$) were determined using an independent samples *t*-test and are indicated by an asterisk (*), while "ns" denotes no significant difference. Data are means ± SE, with $n = 3$.

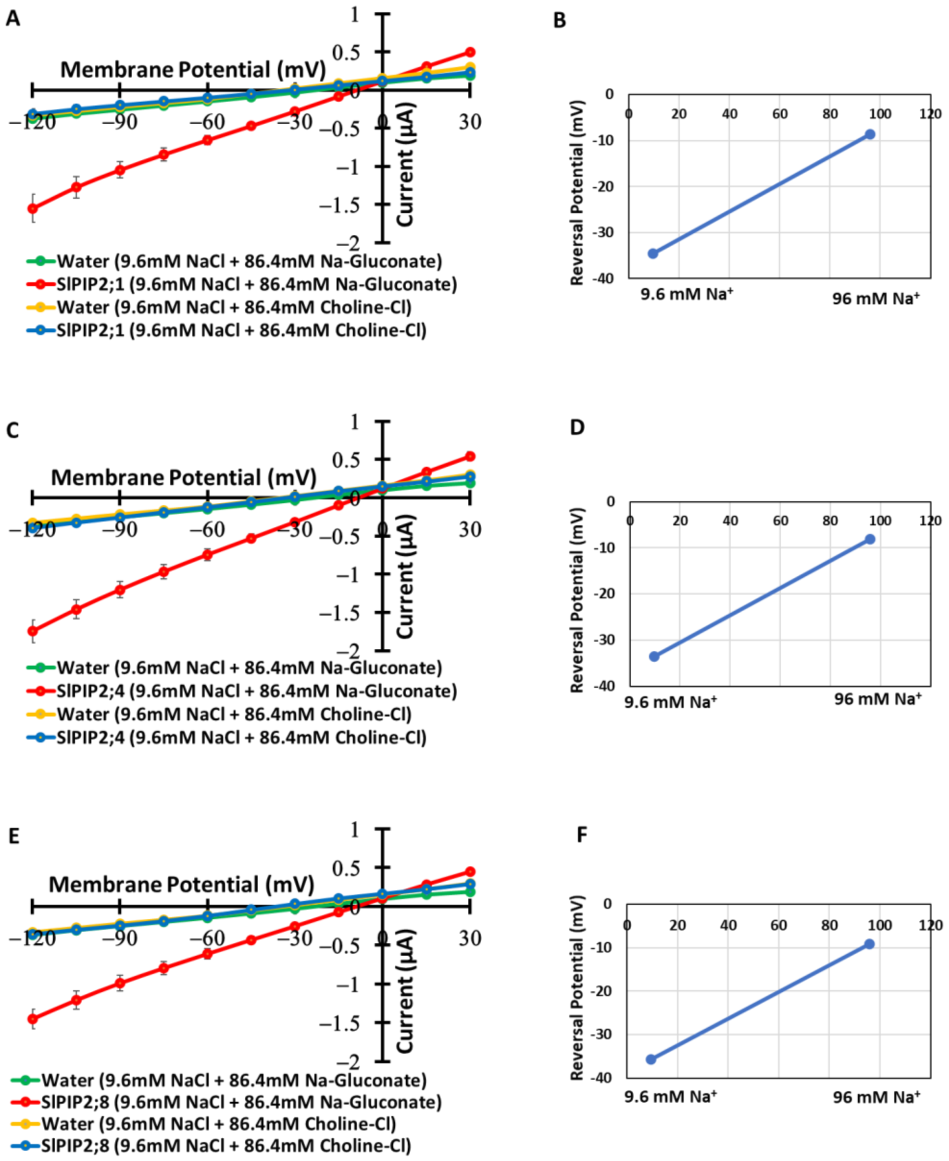

Figure S2. Na^+ transport mediated by SlPIP2;1, SlPIP2;4, and SlPIP2;8 is independent of Cl^-. (**A**, **C**, and **E**) Current-voltage (I-V) relationships were recorded from oocytes expressing SlPIP2;1, SlPIP2;4, or SlPIP2;8, as well as from water-injected controls, in the presence of either 9.6 mM NaCl or 86.4 mM Na-gluconate. (**A**, **C**, and **E**) I-V relationships were also measured in oocytes expressing SlPIP2;1, SlPIP2;4, or SlPIP2;8, or in water-injected controls, under conditions containing either 9.6 mM NaCl or 86.4 mM Choline-Cl. All solutions contained 30 µM Ca^{2+}. *X. laevis* oocytes were injected with 10 ng of cRNA encoding SlPIP2;1, SlPIP2;4, or SlPIP2;8. Reversal potentials were also calculated for SlPIP2;1 (**B**), SlPIP2;4 (**D**), and SlPIP2;8 (**F**). SlPIP2;1, SlPIP2;4, and SlPIP2;8 transport Na^+ but not Cl^-. Data are means ± SE (*n* = 6–9).

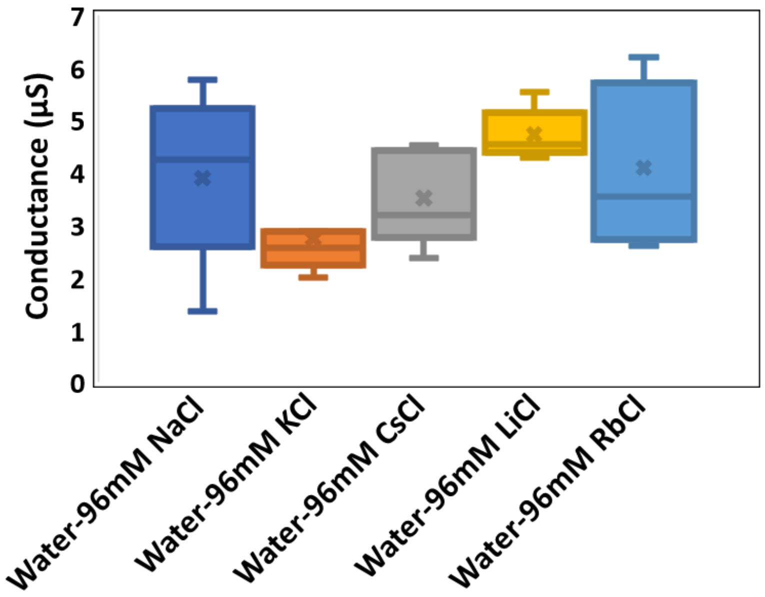

Figure S3. Monovalent cation conductance in water-injected oocytes. Current-voltage (I-V) measurements were obtained from *X. laevis* oocytes injected with water, serving as controls. The oocytes were sequentially bathed in solutions containing low calcium levels (30 µM free Ca^{2+}) and supplemented with 96 mM of either Na^+, K^+, Cs^+, Rb^+, or Li^+ in the form of chloride salts. Ionic conductance was evaluated across a membrane potential range of −75 mV to −120 mV. No significant difference was observed between these five solutions in case of water injected oocytes. Data are means ± SE (n = 5–15).
